# Partial or total knee replacement? Identifying patients’ information needs on knee replacement surgery: a qualitative study to inform a decision aid

**DOI:** 10.1007/s11136-019-02381-9

**Published:** 2019-12-17

**Authors:** Stephanie Smith, Abtin Alvand, Louise Locock, Sara Ryan, James Smith, Lee Bayliss, Hannah Wilson, Andrew Price

**Affiliations:** 1 Nuffield Department of Orthopaedics, Rheumatology & Musculoskeletal Sciences, Botnar Research Centre, University of Oxford, Oxford, UK; 2 Health Services Research Unit, University of Aberdeen, Aberdeen, UK; 3 Nuffield Department of Primary Care Health Sciences, University of Oxford, Oxford, UK; 4 Centre for Healthcare Resilience and Implementation Science, Australian Institute of Health Innovation, Macquarie University, Sydney, NSW Australia

**Keywords:** Knee arthroplasty, Partial knee replacement, Total knee replacement, Information needs, Shared decision-making, Decision aid, Option Grid, Framework analysis, Qualitative

## Abstract

**Purpose:**

For patients with end-stage knee osteoarthritis, joint replacement is a widely used and successful operation to help improve quality-of-life when non-operative measures have failed. For a significant proportion of patients there is a choice between a partial or total knee replacement. Decision aids can help people weigh up the need for and benefits of treatment against possible risks and side-effects. This study explored patients’ experiences of deciding to undergo knee replacement surgery to identify information priorities, to inform a knee replacement decision aid.

**Methods:**

Four focus groups were held with 31 patients who were candidates for both partial and total knee replacement surgery. Two focus groups included patients with no prior knee replacement surgery (pre-surgery); two with patients with one knee already replaced and who were candidates for a second surgery on their other knee (post-surgery). Data were analysed using Framework Analysis.

**Results:**

Participants described a process of arriving at ‘readiness for surgery’ a turning point where the need for treatment outweighed their concerns. Referral and personal factors influenced their decision-making and expectations of surgery in the hope to return to a former self. Those with previous knee surgery offered insights into whether their expectations were met. ‘Information for decisions’ details the practicality and the optimal timing for the delivery of a knee replacement decision aid. In particular, participants would have valued hearing about the experiences of other patients and seeing detailed pictures of both surgical options. Information priorities were identified to include in a decision aid for knee replacement surgery.

**Conclusions:**

Patients’ experiences of surgical decision-making have much in common with the Necessity-Concerns Framework. Whilst originally developed to understand drug treatment decisions and adherence, it provides a useful lens to understand decision-making about surgery. The use of a decision aid could enhance decision-making on knee replacement surgery. Ultimately, patients’ understanding of the risks and benefits of both surgical options could be improved and in turn, help informed decision-making. The knee replacement decision aid is perceived as a useful tool to be associated with other detailed information resources as recommended.

**Electronic supplementary material:**

The online version of this article (10.1007/s11136-019-02381-9) contains supplementary material, which is available to authorized users.

## Introduction

Osteoarthritis is the most common form of knee arthritis in older people aged between 60 and 80 [1]. It is a degenerative condition that can cause joint pain, stiffness, decreased function [2] and can impact both the healthcare system and patients quality-of-life [3, 4]. At the end-stages of knee osteoarthritis, surgery may be recommended when non-surgical treatments are ineffective. Undergoing knee replacement surgery can relieve pain and improve function [5]. In the UK, approximately 100,000 patients per annum undergo knee replacement [6] and globally the numbers performed are steadily increasing.

When patients decide to undergo joint replacement, they may face a choice about the type of joint replacement to be used. Patients are eligible for partial (only the damaged half of the knee is replaced) or total knee replacement when the osteoarthritic disease is localised to one compartment of the knee and the anterior cruciate ligament is intact [7, 8]. Both interventions are standard care. In the UK, approximately 8% of knee replacements preformed are partial [9], although evidence indicates that up to 50% of patients may be eligible [10, 11]. This suggests up to 50,000 patients a year in the UK potentially face a decision between partial or total knee replacement.

Compared to total knee replacement, the partial procedure is less invasive and is associated with a faster recovery, reduced risk of blood transfusion, fewer medical complications and lower mortality [10, 12, 13]. Both procedures significantly reduce pain and improve function for the majority of patients, but comparative evidence suggests that partial replacement is associated with better functional outcome [14, 15]. Yet, long-term studies and registry data show that the revision rate is higher following partial than total knee replacement [12, 13, 16, 17].

Close patient involvement in medical decision-making is now strongly recommended in Europe and the US [3, 18]. Shared decision-making (SDM) is a collaborative process where clinicians and patients share the best available evidence when facing a decision [19, 20], and where patients are supported to consider options, to achieve informed preferences [20]. Whilst being ethically desirable, the wider benefits include better health outcomes and improved patient satisfaction by choosing more appropriate options [21].

Implementing SDM into routine care is difficult despite health policy interest [18]. Mismatches between patients and healthcare professionals have been found in the decision-making process. One study on support needs of patients’ choosing between partial and total knee replacement found patients’ wanted more information on the risks and benefits [22]. However, surgeons were concerned with confusing and overloading patients with information [22]. McHugh and Luker [23] highlighted the need for standardised evidence-based information on knee replacement options. They found many were not provided with treatment options resulting in people searching independently for information and some receiving conflicting advice. Evidence also suggests that some surgeons may not be in equipoise between the two procedures [24]. Considering the different perioperative and post-operative outcomes of partial and total knee replacement, it is likely that these factors will impact patient preference [3].

One way to support SDM is through decision aids, such as Option Grids, providing accurate and accessible information [25]. Option Grids are one-page evidence-based summaries of available options presented in a table format with frequently asked questions (FAQs) listed as the rows derived from common concerns allowing horizontal comparison between options [18]. FAQ answers under each option are based on the latest research evidence [18] and have been developed from patient surveys [e.g. 26] and team decisions (e.g. including clinicians, researchers and patient representatives) based on evidence from patient preference literature [e.g. 27]. They are designed to be read in a few minutes [18] and used in consultations to prompt dialogue [28]. Most importantly, they are developed collaboratively with multi-disciplinary clinical teams and patients [28]. Recent research suggests both patients and clinicians find them acceptable and practical and should be considered within routine consultations [25, 29, 30]. This study consulted the Option Grid guidelines on developing a decision aid.

Greater depth of understanding patients’ concerns/issues may be obtained through qualitative research. The aims of this study were to: (1) explore patients’ experiences before surgery including how they came to decide to have knee replacement surgery, and (2) seek their views on a knee replacement surgery decision aid based on the Option Grid format, including their preferences for content and style of the potential decision aid. Overall, we aimed to uncover information needs/priorities patients need to know to share decisions with clinicians to inform a decision aid comparing partial and total knee replacement.

## Method

Approval was obtained from the London-Surrey Borders Research Ethics Committee and the NHS Research and Development Department in Oxford.

### Participants

Four focus groups were held; two with patients with no prior knee replacement surgery (pre-surgery) and two with patients with one knee already replaced and candidates for knee replacement surgery on their other knee (post-surgery). The number of groups was based on feasibility and gaining insight into pre- and post-surgery experiences. We included pre and post-surgery groups as both groups face the same decision of knee replacement surgery. In particular, the post-surgery groups enabled us to explore their surgery outcomes and review information required post-surgery. All were attending outpatient or pre-operative assessment knee clinics in Oxford. 92 patients were invited: 33 declined, reasons included living a distance away to attend and/or relying on others to bring them, and not feeling comfortable in a group setting; 7 were no longer eligible; 7 were uncontactable; 14 could not attend on the day. 31 patients participated. Table 1 outlines the demographic characteristics.

**Table 1:** Demographic characteristics of 31 patients

Characteristic	Number of participants
Gender	
Male	14
Female	17
Age at focus group	
50–59	5
60–69	14
70–79	9
80–89	3
Duration of symptomatic knee	
Less than a year	4
1–5 years	16
6–10 years	4
10+ years	7
Employment status	
Employed	10
Retired	21
Pre- or post-surgery group	
Pre-surgery	17
Post-surgery	14
Post-surgery group—previous surgery type	
Partial knee replacement	11
Total knee replacement	3

### Recruitment

Purposive sampling was used to recruit participants based on the following criteria: willing and able to give informed consent; aged 18 and above; diagnosed with end-stage knee osteoarthritis; a candidate for either partial or total knee replacement. Participants were excluded if they were a candidate for only total knee replacement or too frail to participate.

Orthopaedic surgeons identified eligible participants by reviewing medical records and introduced SS who explained the study. Interested participants were given the patient information sheet, and contact details exchanged. Focus groups are typically comprised of 5–8 people [31]. To allow for drop-out, we over-recruited [32, 33] and stopped once 10 participants were in each group.

### Focus groups

Focus groups were chosen because they allow for the production of data and insights that may be less accessible without group interaction [34] and are used to explore views on interventions [35]. They are also a platform to inform participants on topics by including experts as co-facilitators, which may lead to better data quality in more complex research contexts [36]. This parallels the growing recognition of integrating researchers, practitioners and patients/consumers in collaborative approaches.

Each focus group lasted up to 2 h, was digitally audio-recorded and held at a research centre. SS, a qualitative researcher and chartered and registered health psychologist was the facilitator. Two orthopaedic surgeons (AA, LB) experienced in both surgical options were the expert co-facilitators and attended two groups each. The surgeons were involved in the presentation of options and when factual information was required or to address queries/concerns. The roles and responsibilities of all group members were outlined at the start of each group and managed by SS who is experienced in conducting focus groups. Consideration of managing the groups was also taken into account in the focus group design. For example, we included background questions to explore the participants understanding of the options before the presentations. We also incorporated an activity-oriented question [33, 37] to discuss around the content to include in the decision aid. This encourages deeper elaboration of ideas and focuses attention [33]. Table 2 outlines the group members characteristics for each focus group.

**Table 2:** Characteristics of the group members

Focus group	Surgery status	Gender	Age range	Duration of symptomatic knee	Employment status	Previous knee replacement
1	Pre-surgery	5 F	50–59 (2)	Less than a year (0)	Employed (2)	–
		2 M	60–69 (2)	1–5 years (5)	Retired (5)	
			70–79 (2)	6–10 years (1)		
			80–89 (1)	10+ years (1)		
Facilitator						
Co-facilitator						
2	Post-surgery	3 F	50–59 (1)	Less than a year (1)	Employed (2)	Partial (6)
		4 M	60–69 (4)	1–5 years (4)	Retired (5)	Total (1)
			70–79 (2)	6–10 years (1)		
			80–89 (0)	10+ years (1)		
Facilitator						
Co-facilitator						
3	Pre-surgery	6 F	50–59 (2)	Less than a year (1)	Employed (5)	–
		4 M	60–69 (6)	1–5 years (2)	Retired (5)	
			70–79 (1)	6–10 years (2)		
			80–89 (1)	10+ years (5)		
Facilitator						
Co-facilitator						
4	Post-surgery	3 F	50–59 (0)	Less than a year (2)	Employed (1)	Partial (5)
		4 M	60–69 (2)	1–5 years (5)	Retired (6)	Total (2)
			70–79 (4)	6–10 years (0)		
			80–89 (1)	10+ years (0)		
Facilitator						
Co-facilitator						

*F* female, *M* male

Number of participants shown in brackets for categories

An independent review of the questioning routes from an orthopaedic patient liaison group (PLG), including patient representatives and multi-disciplinary healthcare professionals, ensured relevance of the questions from a patient perspective. Appendix 1 provides the pre-surgery questioning route and Appendix 2 the post-surgery questioning route.

Focus group structure: Informed consent was obtained followed by a short welcome which included an explanation of anonymity and confidentiality. There were three parts: 1. General introductions; a brainstorming session on what matters most when deciding to have surgery and what is known about knee surgery. 2. Presentation of the options, use of decision aids in the clinical encounter and discussion. The Option Grid on ‘self-management of knee pain, due to arthritis of the knee’ [38] was recommended by the PLG to use as an example. 3. Group work on FAQ’s/information to be included. Participants were split into groups of 2–3 people to discuss the questions and information required for a new decision aid for knee replacement surgery.

### Analysis

Framework Analysis [39] was used to structure and explore the data. This systematic approach addresses specific applied questions for informing policy and practice [39]. NVivo 11 was used to manage the data [40].

The audio-recordings were transcribed verbatim and anonymised. SS led the analysis following the analytical process of the five stages: familiarisation, identifying a thematic framework, indexing, charting, mapping and interpretation. JS was involved in reading and initial coding and differences in interpretation were resolved by discussion. The initial analytical framework was based on the questioning route and aims of the study. The final stage can be a visual presentation of the findings [41]. The coded nodes from NVivo were added to one sheet of paper to review connections [42]. The framework and integration of findings were reviewed by LL and JS. Emerging analysis was also presented regularly to health experience researchers including SR. Final themes were reviewed by all the authors.

## Results

Key and related sub-themes are outlined in Table 3. Throughout this section, we refer to boxes that provide further illustrative quotations from the qualitative data, attached in Supplementary material.

**Table 3:** Key themes and related sub-themes

Theme 1: Readiness for surgery	
*Sub-themes:* Marking time Judging the time—from managing to seeking surgery Time to reflect—expectations and reality	

Theme 2: Information for decisions	
*Sub-themes:* Benefits, choice and recommendations Information timing and needs	

### Readiness for surgery

#### Marking time

Participants often detailed lengthy and complicated journeys to deciding to undergo surgery. Visiting the GP was typically the first step and differences in referral to secondary care were experienced. Difficulties ‘getting to see the surgeon’ as Samuel described, and going through a series of non-surgical interventions including pain medication, physiotherapy and steroid injections before considering surgery were common (Box 1 in Supplementary material). Many became frustrated with their GPs, and other avenues of care were now perceived as what Floyd called ‘a delaying tactic’ for surgery.

For some, personal circumstances including family ill health prevented them from proceeding with surgery (Box 2 in Supplementary material). Knee osteoarthritis is not life threatening and personal delays experienced suggests timing of surgery was viewed as flexible.

#### Judging the time—from managing to seeking surgery

Initially, for some, adapting their movement (e.g. using devices to pick-up items) prevented knee symptoms dictating their life. Some found creative ways to accommodate knee problems rather than accept surgery, Ellen described: ‘I’ve been coming downstairs backwards for years.’ However, others like Molly feared delaying surgery: ‘I want to go ahead…before I kind of get to that point.’ There could be an urgency for those with knee replacements to get the second done for concerns of putting ‘pressure’ (Grace) on the replaced knee and damaging the prosthesis.

Many described becoming housebound, stopping activities, relying on pain medication and feeling unsafe. The frustration of not being able to continue with activities affected their identity. A common concern was feeling ‘old before my time’ (Doug) and missing out socially was another concern. Surgery provided an opportunity to resume a former self and ‘get back to normal’ (Rose).

A turning point was often described, ‘I was in such pain that really I didn’t care about what the implications were’ (Lloyd). Risk factors were outweighed by the hope of removing pain, stiffness, swelling, and improving posture, mobility, control and quality-of-life. Surgery offered an opportunity to gain their life and control back (Box 3 in Supplementary material).

For some, judging the time for surgery was assisted by routine questionnaires at surgical appointments. Questionnaires such as the Oxford Knee Score (OKS) [43] that rates pain and function were found to be ‘incredibly helpful’ (Olivia) and prompted participants to proceed with surgery (Box 4 in Supplementary material). However, many queried the OKS noting the score appeared on their post-appointment letter. Once explained, many would have found this information beneficial in assessing their decision and outcome.

#### Time to reflect—expectations and reality

The post-operative group largely discussed quick and satisfied outcomes from surgery, with some dissatisfaction when participants were unable to return to activities or experienced long recoveries. Most were pleased they were eventually pain free with improved mobility and wanted to move forward with the next surgery on their other knee (Box 5 in Supplementary material).

Post-operatively, most experienced what Tim described as ‘a sense of abandonment.’ Information was needed on exercises they should be doing post-operatively (and pre-operatively, this was also mentioned by the pre-surgery group) to aid fitness and recovery. Kneeling was a frequent concern (this was also a concern for the pre-surgery group) and what could and should not be done to prevent harming the prosthesis. They also wanted more information on pain management and dealing with medication side-effects such as constipation.

### Information for decisions

#### Benefits, choice and recommendations

The decision aid was received as a useful tool. Participants talked about how it could make them feel ‘forearmed’ (William) and provide them with a ‘starting point’ (Celia) for further research, and some reflected how it would help the consultation and act as an information resource (Box 6 in Supplementary material). One participant did not like the proposed tool: ‘I’m not the sort that bothers about things like that…but…I can see that it’s good for a lot of people’ (Stewart).

The presentation and discussion of surgical options made some realise they had not fully understood the options (Box 7 in Supplementary material) and having a decision aid would have made them better informed.

However, whether patients had a choice on which knee replacement caused some debate. Some argued the decision should be down to the surgeon and wanted to know the surgeon’s preference. Others felt involved and given a choice, with some reporting that the decision was a surgical one (Box 8 in Supplementary material). However, despite the clinical aspect, some argued they still had a choice by presenting their ‘preference’ (Eric), and the tool would prepare them on the possible outcomes.

Participants discussed recommendations to the tool. Information presented concisely was essential and ‘bullet points’ (Tim) were suggested for clarity. Other recommendations included a space for notes.

Several people suggested quantifying the information—‘putting a number on it’ (Dean)—would help them understand and weigh up the pros and cons of the options.

Others wanted more qualitative information about patient’s experiences (Box 9 in Supplementary material). Including quotations from people’s stories on one side of paper was recognised to be difficult due to space. Instead, it was suggested that the tool should be part of a wider information resource.

#### Information timing and needs

The appropriate timing to present the decision aid which would determine the FAQ’s and information needs was discussed. Participants imagined themselves in various scenarios and the effect of receiving the tool before seeing the surgeon:

won’t you send that out to people who have knee pain…that’s not due to arthritis…they may say, “Oh but in there it says I can have a partial knee replacement, and actually you’re telling me no” (Gayle)

It was recommended that the tool should be presented at the patient’s appointment and the surgeon was best placed for the conversation on surgery options rather than a GP or physiotherapist:

when I first came in, I knew about knee replacements, but I had no idea or no knowledge of what the procedures were (Patrick)

The information needs will now be discussed under the following subheadings. Table 4 provides a summary of the information priorities.

**Table 4:** Summary of the information priorities identified

Information needs and concerns	Examples
Background to the options	What the surgeries involve Physical differences
Outcomes	Relief in pain, long-term pain relief Function improvements (e.g. mobility, kneeling) Survival of prosthesis/failure (revision) Further treatment (e.g. rehabilitation/physiotherapy) Complications (e.g. infection, blood clots, nerve damage)
Cosmetic	Shape of knee Scar concerns
Time	Age concerns for surgery Length of procedure Recovery (e.g. length of stay in hospital/return to activities/sports)

#### Background to the options

A key aspect to the new decision aid was knowing what surgery involves:

I’d want to know is what is the difference between the two [options]…so I need a definition of what one is as opposed to the other (Eric)

Detailed diagrams/pictures of both options were recommended to be provided on the reverse of the tool.

#### Outcomes

Information on outcomes included needing to know about the improvements knee replacement could provide:

The first one was outcomes in terms of long-term pain relief…improved mobility (Gayle)

Having information on the prosthesis outcomes was also important (Box 10 in Supplementary material).

Further treatment post-surgery was also questioned:

Do you need…extensive physio post-op? (Anna)

Many stated the importance of having the ‘medical complications that can happen’ (Patrick) during the operations. They drilled down further highlighting specific risks such as blood clots, nerve damage and infection.

#### Cosmetic

Cosmetic improvements to their knee were queried. Tim described:

I used to be comfortably over six feet, but I’m not any more…your legs go like a jockey’s…you walk around in your best suit…people say, “What’s happened to your leg sort of sticking out at a funny angle?”

Many enquired about the length of the scars and their visibility. Katie had a good outcome, but she reflected on meeting others which had caused her worry:

a chap…had two partial knees…one scar was quite okay…white and the other one was zigzag and red…he said “This one was infected.”

Having ‘less scar tissue’ (Doug) made some consider their choice.

#### Time

Time was a factor in terms of age, length of procedure and recovery. Particularly, older patients questioned their age and undergoing surgery.

Many were surprised that the duration of both procedures was shorter than expected. Some remarked that the length of the procedure would not impact their decision, however for Karl it did: ‘The less time you’re going to be out the better.’

What is meant by recovery also needs to be clarified:

recovery to do what?…have a shower…drive cars…walk a mile…ride a bike five miles. (Gayle)

For the majority, recovery meant time in hospital and getting back to physical activities.

## Discussion

Participants arrived at ‘readiness for surgery’, a turning point where accommodating their condition gave way to a perceived need for surgery and hopes of improved function as a result. ‘Information for decisions’ included the tool to be presented by the surgeon, suggested benefits and recommendations, views on choice and important information priorities identified.

In the current study, many experienced difficulties being referred to secondary care and became frustrated with their GP, perceiving other treatments as hindering surgery. McHugh et al. [44] found those with worse pain and physical functioning were more likely to have knee replacement surgery. They suggested improvements are required to GP referral processes and guidance on who should have surgery. The ACHE (Arthroplasty Candidacy Help Engine) Tool is a new referral guide using the OKS to identify candidacy for joint replacement [4]. User evaluation revealed the tool was viewed positively by patients and GPs to support referral but low response rates are noted [4]. In this study, there was support for the ACHE Tool to guide referral decisions and improve patients experience of the referral process.

Readiness for surgery was based on a number of factors including pain with a desire to stop pain medication, improve activity levels, financial concerns and psychosocial improvements. The Necessity-Concerns Framework (NCF) provides a useful lens through which to interpret our findings and its theoretical implications has been suggested to extend to surgery [45]. The NCF was developed to understand attitudes to medication and helps explain the relationship between common-sense evaluation and extent of treatment adherence [46–48]. Adherence is influenced by perceptions of the need of treatment (necessity beliefs) and concerns about the adverse effects of that treatment (concern beliefs) [47].

In the current study, perceptions of personal need were often masked by normalising and adapting movements (a finding also noted by Hudak et al. [49]) preventing a need for treatment. Andersen et al. [50] refer to this as ‘appraisal delay’ when delays are encountered due to symptom interpretations.

Participants traded-off costs and benefits of surgery. Concerns consisted of kneeling issues post-surgery and surgery complications (e.g. infection/recovery). A threshold was hit when other alternatives were exhausted, a finding noted by Suarez-Almazor et al. [51] in their study on total knee replacement surgery decision-making. Often experienced was an interaction of biopsychosocial factors [52]. Biologically, the knee was becoming uncontrollable/unpredictable and painful. Psychologically, identity was impacted (e.g. feeling old) with a need to gain control back (e.g. regaining mobility) (see also [23, 51]). Many participants were missing out on social activities. Smith et al. [53] found social isolation through reduced participation and functional capability. In the current study, surgery was eventually associated with stronger perceptions of necessity for treatment and fewer concerns about adverse consequences in the hope of returning to a former self.

Our study supports previous findings that decision aids are acceptable tools [25]. Despite variation on surgery choice, most wanted an active role in SDM and the surgeon’s recommendation. Similar findings in orthopaedics and cancer care have been found [54, 55]. Woolhead et al. [56] found participants struggled to make sense of their outcome of knee surgery and often described it in contradictory terms. In the current study, participants felt the decision aid would prepare them for all possible outcomes.

Recommendations included using the back of the page for images and notes. Previous research has supported the use of images on decision aids and has often been used to bridge literacy barriers [48, 57]. We recommend visual information should not be limited to literacy level.

Further recommendations to the tool included using bullet points for clarity and a quantitative format which falls into the traditional didactic approach of health information presented by facts and statistics [58, 59]. However, the benefits of hearing others experiences were noted in the current study. Patient narratives have been increasingly used to provide health information to patients [58–60]. Narratives are easily processed [59, 61] and may also provide important emotional and social information often lacking in health resources [59]. We recommend that the knee replacement surgery decision aid will be a useful adjunct to the clinical discussion and needs to be part of a whole that includes patient experience and pre/post-operative information to better support patients in their decision-making. In support, Bennett et al.’s [59] study found a combination of supplementing factual information with patient narratives was useful and likely provided better understanding of cancer screening.

The findings support existing literature around the importance on outcomes (e.g. pain reduction/function/flexibility/kneeling/mobility [e.g. 5, 14, 15]), risks (e.g. blood clots/nerve damage/infection/survival of prosthesis/rates of revision [e.g. 12–17, 62–64]) and time factors (e.g. age/length of procedure/recovery/time in hospital [e.g. 13, 14, 49, 65–70]). It further identifies original priorities, such as cosmetic concerns including the appearance of the knee and scar concerns. Research has found satisfaction with limb alignment appears to influence outcome after total knee replacement and is often excluded in outcome measures [71] and that high concerns about scarring following elective surgery are irrespective of age, gender, ethnic background or geographic location [72]. Expectations need to be addressed between patients and clinicians [71, 72] which we envision with the implementation of this decision aid.

### Study limitations

Participants were recruited from one hospital and were white and English speaking, which may limit the applicability of the findings. It is important to conduct research with a demographically diverse sample. Having orthopaedic surgeons involved in the focus groups may have deterred some participants. The post-surgery group had more partial than total knee replacement participants. However, the post-surgery group were in the same situation as the pre-surgery group faced with options which many did not experience with their first knee replacement.

### Study outcomes and future directions

Our findings highlighted the need for a decision aid focused on partial and total knee replacement. A reference group was initiated, the study findings consulted on, FAQ’s determined and information prioritised to include in the decision aid. A systematic review has been completed comparing partial versus total knee replacement to help inform the FAQs [73]. The tool will be user-tested and evaluated in the clinical setting.

## Conclusions

Patient decision-making for knee replacement surgery could be improved by the use of a decision aid to help people weigh up their concerns against their need for surgery and potential benefits. This would improve patient understanding of the risks and benefits of both surgical options and help patients make an informed decision. It is recommended that information on patient narratives and pre- and post-operative information (e.g. exercises to aid recovery) would supplement the decision-making.

### Electronic supplementary material

Below is the link to the electronic supplementary material.

Supplementary material 1 (PDF 497 kb)

## Appendix 1: Pre-surgery questioning route plus prompts

**Table:**

*Part 1*	
Opening question	1. Let’s start by introducing ourselves. Could you inform the group of your name, a little bit about yourself and fill in the blank with any word that comes to mind, ‘My knee is ____________’
Brainstorming session	2. What matters most to you when deciding whether to have knee replacement surgery? Prompts: Issues or concerns/recovery/activities (e.g. housework, exercise) *Facilitator/co-facilitator to write comments on the board/flip chart*
Background questions	3. What do you know about your surgery so far? Prompts: Information given/risks/benefits/options provided? 4. Imagine yourself one year after surgery. What would have to be different about your life for you to say it was worth it? Prompt: What are you hoping for?
*Part 2*	
Presentation	• Presentation on the two options • Show an example of an Option Grid—highlight 6–8 FAQs, evidence-based answers • How Option Grids are recommended to be used in the medical encounter
Key questions	5. You have all been invited today because you are all possible candidates for either a partial or total knee replacement. What are your thoughts on these two options? Prompt: Have any of you discussed the two options with your surgeons? [Link in with question 3]. 6. What are your thoughts on Option Grids? Prompts: Do you think they are useful?/likes/dislikes of the Option Grid/level of information required in the Option Grid/how the Option Grid is suggested to be used. 7. How do you feel about being actively involved in the decision of which knee replacement to have? Prompts: Issues/concerns/empowering? 8. Do you think the surgeon should tell you if they have a preference on which surgery (partial or total) you should have?
*Part 3*	
Group work	9. What information/questions do you think needs to be included in the new Option Grid on partial and total knee replacement surgery options to treat knee osteoarthritis? Prompts: What are the most important questions that need to be listed?/What information is required to aid decision-making between partial and total knee replacements? • *Comments from brainstorming session to refer to* • *Flip chart paper and pens available to participants.* • *Groups will come together as a whole to review lists/comments.*
Ending questions	10. Of all the things we’ve discussed today, has anything come up that has surprised you? 11. Is there anything else we haven’t discussed yet that you think is important to know about before we develop the Option Grid? Prompt: Is there anything that you wanted to say but didn’t have the chance to?

## Appendix 2: Post-surgery questioning route plus prompts

**Table:**

*Part 1*	
Opening Question	1. Let’s start by introducing ourselves. Could you inform the group of your name and fill in the blank with any word that comes to mind, ‘My knee is ____________’ This could be on your current knee or the knee to be operated on, or both.
Brainstorming session	2. Thinking back to before you had knee replacement surgery, what mattered most to you when deciding whether to have knee replacement surgery? Prompts: Issues or concerns/recovery/activities (e.g. housework, exercise) *Facilitator/co-facilitator to write comments on the board/flip chart* 3. Thinking back is there any information you’d wished you’d know about before your knee replacement surgery? 4. Some of you are shortly undergoing knee replacement surgery on your other knee or this is planned for the near future, have your views changed on what matters to you since having knee replacement surgery?
Background questions	5. What information were you or have you been told about your surgery so far? Prompts: Information given/risks/benefits/options provided? 6. Thinking back to before you had knee replacement surgery, what were your expectations? Prompts: What are you hoping for?/Did the reality meet your expectations? 7. What are you hoping for with your upcoming knee replacement surgery or would you be hoping for with future knee replacement surgery?
*Part 2*	
Presentation	• Presentation on the two options • Show an example of an Option Grid—highlight 6–8 FAQs, evidence-based answers • How Option Grids are recommended to be used in the medical encounter
Key questions	8. You have all experienced one of these options (partial or total) and some of you may shortly be having knee replacement or in the near future on your other knee, what are your thoughts on these two options? Prompt: Have any of you discussed the two options with your surgeons? [Link in with question 5]. 9. What are your thoughts on Option Grids? Prompts: Do you think they are useful?/likes/dislikes of the Option Grid/level of information required in the Option Grid/how the Option Grid is suggested to be used. 10. How do you feel about being actively involved in the decision of which knee replacement to have? Prompts: Issues/concerns/empowering? 11. Do you think the surgeon should tell you if they have a preference on which surgery (partial or total) you should have?
*Part 3*	
Group work	12. What information/questions do you think needs to be included in the new Option Grid on partial and total knee replacement surgery options to treat knee osteoarthritis? Prompts: What are the most important questions that need to be listed?/What information is required to aid decision-making between partial and total knee replacements? • Comments from brainstorming session to refer to • Flip chart paper and pens available to participants. • Groups will come together as a whole to review lists/comments.
Ending questions	13. Of all the things we’ve discussed today, has anything come up that has surprised you? 14. Is there anything else we haven’t discussed yet that you think is important to know about before we develop the Option Grid? Prompt: Is there anything that you wanted to say but didn’t have the chance to?
