# Supplementary material for: Partial or total knee replacement? Identifying patients’ information needs on knee replacement surgery: a qualitative study to inform a decision aid
Source: Qual Life Res. 2019 Dec 17;29(4):999–1011. doi: 10.1007/s11136-019-02381-9 (PMC7142054; doi:10.1007/s11136-019-02381-9)
Supplement: Supplementary file 1 — Supplementary material 1 (PDF 497 kb) [file 11136_2019_2381_MOESM1_ESM.pdf]

Supplementary material

Partial or total knee replacement? Identifying patients' information needs on knee replacement surgery: a qualitative study to inform a decision aid

Box 1 **Non-surgical interventions**

Dean

I went to see my GP he basically said to me, "you've got to go and have physio before I can do anything else." So, I had six weeks of very painful physio, went back to the doc and he said, "OK I'll fix you up and go to the [hospital] and see the consultant."

Lillian

It took me a long time to get where I wanted to get…my GP, he palmed me off with painkillers and possibly physio…which I've tried and been there, done that…then I had the luck. I actually see…a locum doctor…I explained it all to her. "Oh, no, no, no," she said, "well, I'll try and get you sorted out."

Box 2 **Personal circumstances preventing surgery**

Mack

With not knowing whether it's [surgery] going to be three months or six months, it totally can disrupt your whole life and your life with everybody around you because you're not sure if you could book a holiday…I would rather be in denial and just carry on.

Doug

I'm…self-employed, and it's a very, very seasonal business and it starts to get ridiculously busy this time of year after almost three months of virtual unemployment…I did wonder whether if I had it later on in the year when it [occupation] starts to go quiet.

Jesse

I was actually supposed to have a knee replacement three years ago but my health wasn't good enough.

Celia

my husband…he's been through the cancer and the ops and all that sort of stuff…I've just had my knee done [after several years]. We had to cancel it first time round, because...his problems overtook anything, you know, so my knee was kind of irrelevant.

Box 3 **Gaining life and control back through surgery**

Patrick

Your own way of life, it improves…One, it's compound because…once I can walk properly, and I can walk upright again, that will improve my posture, it'll improve a lot of other things and…begin to live and accept and enjoy a normal life. Because I don't enjoy it, I struggle.

Box 4 **The Oxford Knee Score (OKS) aiding the decision**

Olivia

My whole life was actually understood by the questions that were on there [OKS].

Lloyd

The optimum is forty-eight [OKS], and my left knee…was twenty-five…a knee that hasn't been touched before you would hope to get a twenty-unit improvement, so that brings me up to forty-five, which is very near the optimum, so I thought, "yeah I'll go ahead."

Box 5 **Outcomes of surgery**

Floyd

The benefits I received from the first operation [partial] were unbelievable…I was in hospital and went home the next day, within a fortnight I was walking unaided, no crutches and it was absolutely super. And I can't recommend it highly enough…[Before surgery] Couldn't sleep at night. Was sleeping with one leg copped over a pillow and all the rest of it.

Celia

My right knee [partial] doesn't like riding all that much.

William

It took a long time to get better [total]. I went back for my six-month check-up, and about a month before I thought "I really wish I hadn't had this done," it was so painful. And literally a fortnight before I went to see him [surgeon] suddenly, magic, it got better.

Grace

Well it just helped me when I had it done [partial] and afterwards, I just couldn't believe that I could get about with not having the pain that I was having before, and to go up and down the stairs was better than what I was in before, because just doing one step at a time took me ages to get up and down the stairs. But now that's a lot better but I'm having to do, now, one at a time because of my other knee, so I'm back to square one again. I'm just waiting now for this one to be done, yeah.

Box 6 **The decision aid as an information resource**

Olivia

Although there were two of us there, we didn’t both hear everything that was said, so having something written would be immensely helpful I feel.

Laura

It will give you ideas, you know for questions, you know to ask the surgeon. I wish we'd have had it with the first knee…because it’s quite difficult…you come out of a consultation and then you think, “oh well I wish I’d have said that or that,” you know.

Doug

Having it in black and white and something that you can read is, it's so much better than in a verbal form, that you can sort of miss the interpretation or something like that, or you'll not get it at all, so, and having something that you can refer to later and just assimilate it.

Katie

Because a lot of people that are eligible for these operations may not be computer literate, and won’t be able to look up on Google or whatever what the operations entail. And so, having the information on a grid, and the ease of sitting to discuss it with a professional, I would have thought could only be a good thing.

Box 7 **Misconceptions of surgery**

Dean

When I was vetted and they said a half-knee, I was slightly disappointed. I thought, “ah being done on the cheap here.”

Molly

I didn’t realise it was like cement or anything, I was just thinking it was a plate and was screwed in together.

Box 8 **Choice**

Anna

I've said to my consultant he's the expert, so I'll leave it up to his final decision.

William

Definitely given a choice but with a recommendation.

Tim

I would have a partial, but that it may turn out to be a full.

Box 9 **Qualitative information**

Mack

But speaking to people who'd actually been there, done it, it'll be great to have what they wrote about what they thought the best advantages were; how they feel afterwards.

Box 10 **Prothesis outcomes**

Samuel

I think some information on how long the bloody thing lasts and likely to see you out. The chances of failure.

Gayle

What percentage needed revision; think that would be useful, because if I was told it's only going to last five years, I'd think again with what I had done. If you tell me it's going to last twenty-five, that's fine.
